# Multi-Scale Characterization of the PEPCK-C^mus^ Mouse through 3D Cryo-Imaging

**DOI:** 10.1155/2010/105984

**Published:** 2010-05-11

**Authors:** Debashish Roy, Madhusudhana Gargesha, Grant J. Steyer, Parvin Hakimi, Richard W. Hanson, David L. Wilson

**Affiliations:** ^1^Department of Biomedical Engineering, Case Western Reserve University, Cleveland, OH 44106, USA; ^2^Department of Biochemistry, Case Western Reserve University, Cleveland, OH 44106, USA; ^3^Department of Radiology, University Hospitals of Cleveland, Cleveland, OH 44106, USA

## Abstract

We have developed, for the Case 3D Cryo-imaging system, a specialized, multiscale visualization scheme which provides color-rich volume rendering and multiplanar reformatting enabling one to visualize an entire mouse and zoom in to organ, tissue, and microscopic scales. With this system, we have anatomically characterized, in 3D, from whole animal to tissue level, a transgenic mouse and compared it with its control. The transgenic mouse overexpresses the cytosolic form of phosphoenolpyruvate carboxykinase (PEPCK-C) in its skeletal muscle and is capable of greatly enhanced physical endurance and has a longer life-span and reproductive life as compared to control animals. We semiautomatically analyzed selected organs such as kidney, heart, adrenal gland, spleen, and ovaries and found comparatively enlarged heart, much less visceral, subcutaneous, and pericardial adipose tissue, and higher tibia-to-femur ratio in the transgenic animal. Microscopically, individual skeletal muscle fibers, fine mesenteric blood vessels, and intestinal villi, among others, were clearly seen.

## 1. Introduction

The availability of the mouse genome sequence and advances in genetic manipulation has resulted in multiple initiatives, both in the public and private sector, to produce mouse mutants on a large scale. Coordinated efforts are on way to systematically knock out all mouse genes to provide researchers targeting constructs, vectors, live mice, and phenotypic data at a variety of level and detail [1]. The relationship between the effect of genetic manipulation and the resulting phenotype is a key component to establishing a fundamental understanding of molecular and cellular process [2]. Parallel to the growth in mouse models to study disease patterns, rapid strides have been made in developing imaging instruments specifically targeted to the small animal. Instruments like MRI, PET, SPECT, CT, in vivo fluorescence, in vivo bioluminescence, and intravital imaging allow one to visually, and sometimes quantitatively, evaluate biological processes at the cellular and subcellular level in a living subject [3]. However, the major challenge in successfully characterizing morphological features of transgenic mouse models has been the trade-off between resolution and field of view. Researchers particularly want the ability to detect gross structural change or, in the absence thereof, localize on subtle variations at microscopic level in 3D in tissue and cell structures. Thus the ideal system used in characterizing transgenics should offer multiscale 3D visualization at a very high native resolution. In this paper we describe the application of cryo-imaging system and the multiscale approach to characterize a transgenic mouse.

Our group has developed a cryo-imaging system, which provides contrast rich, bright field anatomical, and fluorescence cellular and molecular imaging of an entire mouse with micron-scale resolution. The system acquires three-dimensional (3D), very high-resolution, large field of view (FOV), brightfield anatomy and fluorescence molecular image volumes from sequential images of the tissue block face by alternately sectioning and imaging. In an earlier report, we have demonstrated few applications of cryo-imaging ranging from high-resolution anatomical imaging of whole mouse, vascular imaging, and molecular fluorescence imaging of fluorescent mouse models and embryos [4]. The system has been used for validation of MR studies of human blood vessel lesions [5], ex vivo characterization of human atherosclerotic iliac plaque [6], and very recently for single cell detection of fluorescently labeled cancer and stem cells [7]. True color enhanced volume rendering techniques provide fast 3D visualizations of cryo-imaged samples [8].

Cryo-imaging is unique among all 3D in vivo (e.g., micro-MRI) and microscopic techniques, because it allows micron resolution and information-rich color contrast mechanisms over very large 3D fields of view. MRI imaging of whole mouse can produce gray-scale images after administering contrast agents with significantly less resolution [9]. Optical Projection Tomography is limited to small samples such as embryos and requires treatments to reduce scatter and increase transparency [10]. Diffuse optical tomography while tackling the light scatter problem with advanced algorithms [11] can image a whole mouse albeit at limited resolution providing little anatomical detail. Johnson et al. used very high-resolution CT and osmium tetroxide staining to image tissues and embryos [12]. Serial histology techniques to create a 3D volume involve serially cutting the sections, applying histological processing, mounting on glass slides, digitizing the slides, and then creating a 3D volume from the two-dimensional (2D) images. Such systems have been used for a variety of biomedical applications [13, 14] including characterizing phenotypical change in large histological mouse model datasets [15]. However, such processing suffers from tears, tissue shrinkage, errors in image alignment, and typically time-consuming manual editing of 3D reconstructions. A block-face imaging system like ours alleviates much of these difficulties. Systems have been mostly applied for imaging excised organs such as brains [16, 17] and embryos [18, 19] with adult whole mouse block-face imaging being limited to low-resolution photographs of sections taken for autoradiography studies [20] and CT and block-face imaging for creating a mouse atlas [21].

We have developed the robotic 3D cryo-imaging system and first applied it to high-resolution tiled imaging of a wild-type mouse. Now we report, for the first time, morphological characterization of a transgenic mouse recently generated by researchers at Case Western Reserve University [22]. This mouse, dubbed “supermouse” by popular press, contains a chimeric gene in which the cDNA for PEPCK-C was linked to the *α*-skeletal actin gene promoter, expressing PEPCK-C in skeletal muscle. The transgenic PEPCK-C^mus^ mice were seven times more active than their controls in their cages, ran for up to 5 km at a speed of 20 m/min on a mouse treadmill (control animals run for 0.2 km at this speed). As reported earlier, the mice eat almost twice as much as controls but are half the body weight; they also have an extended life span relative to the controls [22]. In their seminal report, the researchers have detailed the study of biochemical pathways to better understand the repatterning of energy metabolism. The battery of tests involved assay of metabolites in blood and tissues, home cage activity testing, exercise capacity testing, respiratory quotient (RQ) measurements during exercise, and histological and electron microscopy analysis of skeletal muscle.

Characterization of the PEPCK-C^mus^ mouse with a cryo-imaging, multiscale approach involves detailed high-resolution imaging of a whole mouse and a control mouse, processing and manipulations of enormous giga-byte sized data volumes, and enhanced 3D visualizations. In the following sections we describe the cryo-imaging setup, the imaging techniques, and enhanced visualizations in 3D at multiscale and qualitative and quantitative comparison of organs.

## 2. Cryo-Imaging System

The cryo-imaging system is described in detail in our earlier report [4]. Briefly, it consists of a cryo-microtome, a microscope imaging system, a robotic *xyz* positioner, and a computer control system. The cryo-microtome is a motorized, large section, whole body cryo-microtome with section thickness adjustable between 2–40 *μ*m and a maximum specimen dimension of 250 mm × 110 mm × 50 mm. An *XYZ* robotic positioner carries the imaging system comprising of a stereo microscope, coaxial fluorescent attachment with multiple filter cubes, low light digital camera, and brightfield and fluorescent light sources. The computer control system automatically pans the positioner over the specimen for a high-resolution tiled image acquisition and controls the sectioning and image acquisition sequence through the custom developed Programmable Sectioning and Cryo-Imaging (ProSCI) software. The Image Processing and Visualization system is a quad-core Windows 64-bit PC with 32 GB of RAM capable of handling large cryo-image volumes. A suite of MATLAB custom programs and custom AMIRA scripts are used for image preprocessing tasks and 3D visualization.

## 3. Methods

### 3.1. Sample Preparation and Imaging

In order to acquire cryo-image data, mice covered under IACUC-approved projects, were euthanized in a method approved by Case Animal Resource Center which consists of either inhalation of carbon dioxide delivered from tank or anesthetization using an agent such as pentobarbital, at a dose prescribed by the Case ARC. A cryo-embedding compound, OCT (Optimal Cutting Temperature, Tissue-Tek, Terrance, CA), was used to completely immerse the mouse in an aluminum foil mould. The mould was then covered in more foil and placed inside a freezing chamber made of styrofoam and filled with liquid nitrogen. After about 15 minutes, the mould assembly was removed from the liquid nitrogen bath and placed inside the cryo-microtome chamber to equalize the specimen temperature to the cryo-microtome temperature. The mould was removed from the specimen after two to three hours and the frozen mouse block was mounted on the microtome stage using more OCT. An initial “facing-off” cycle continuously sliced the block at maximum thickness until animal tissue become visible. Then the section thickness was set to 40 *μ*m, the imaging system was readied for a pixel size of 15.6 *μ*m, and the system was started to alternately slice and acquire tiled brightfield images of the block-face. The system was run completely unattended with the user periodically alerted by the ProSCI program about imaging progress through email and cell phone text messages. Female PEPCK-C^mus^ and control mice were chosen for cryo-imaging. Details of the transgenic mice are available elsewhere [22]. The transgenic animal used in this study weighed 25.8 gm and was 421 days old when sacrificed. The control mouse weighed 37.8 gm and was 375 days old.

### 3.2. Image Acquisition

Once the mouse specimen was mounted on the stage, the operator selected the boundaries of the acquisition volume and the system calculated the number of tiles required per block-face taking into account the operator-entered minimum overlap zone between adjacent tiles, typically 15%. The robotic system computed the coordinates of each tile based on the pixel size and automatically sectioned and imaged the entire volume. Brightfield images (each 1360 by 1036 pixels) were acquired in 6 × 4 tiling pattern to cover each block-face. A total of 586 sections were needed for the transgenic mouse and 616 for the control. A metadata file stored the global coordinates, imaging parameters, and experiment parameters.

### 3.3. Image Processing

Following acquisition of 2D images on the imaging workstation, nonuniform illumination pattern was compensated using a reference image of a white card and compensated images for each tiled block-face were composited together to yield a typical 2D whole mouse section image of 6500 by 3600 pixels. These stitched images were then automatically aligned to each other to correct for minor misalignments due to the repositioning error of the stage at the return point. The total stitched 2D image stacks amounted to a data size of >60 GB. Since such a large dataset cannot be loaded fully in the memory of the visualization workstation, we converted the stack of images into a Large Data Access (LDA) volume inside our customized AMIRA Visualization software. LDA allows us to subsample at various resolutions an entire mouse or organ, or to sample the data at full resolution within smaller cropped subvolumes. From a relatively low-resolution 3D mouse, subregions could be interactively selected for high-resolution viewing.

### 3.4. Enhanced Volume Visualization

The whole mice were segmented from the OCT blocks through our fully automated volume visualization. Since cryo-image data is in color, unlike other gray-scale medical imaging data (MRI, CT, etc.), this color information can be exploited. We have designed color feature detectors which take into account the dominant color of a region to segment it. Examples of red, green, and blue ratio feature detectors (*f* *R*, *f* *G*, *f* *B*) are shown below, where *R*, *G*, and *B* refer to the 8-bit data for red, green, and blue channels, respectively,

(1) $$\begin{array}{c} fR=\frac{R}{R+G+B},\;\;fG=\frac{G}{R+G+B},\\ fB=\frac{B}{R+G+B}. \end{array}$$

For instance, regions containing a high proportion of red such as liver, lungs, and vasculature can be extracted using the red feature detector *f* *R*. For detecting stomach and intestinal regions, which are predominantly brown in color, we exploited the fact that brown is composed of one part of *R*, two parts of *G*, and zero (0) parts of *B*. A brown feature detector is therefore expressed as a weighted linear combination of red and green feature detectors:

(2) fBr=(0.33fR+0.67fG).

For segmenting out the embedding compound OCT, which has a uniform white color a balanced color detector was used:

(3) $$\begin{array}{cc} f & =\frac{\left[\text{abs}\left(R-G\right)+\text{abs}\left(G-B\right)+\text{abs}\left(R-B\right)\right]}{3}*I_{\max\;},\\ & \text{where}I_{\max\;}=255. \end{array}$$

Subsequent to feature detection, opacity transfer functions (OTFs) were used to assign an appropriate *α* opacity value to each voxel. Below, we show an example of color-based step OTF that we have employed in our visualizations:

(4) $$\alpha =\{\begin{array}{cc} 255, & f>T,\\ 0, & f\leq T, \end{array}$$

where *T* is an empirically determined threshold. In other algorithms, we have optimized opacity by imposing several simultaneous constraints on an opacity function which maps each voxel to a custom opacity value. Enhanced volume rendering techniques for high-resolution color cryo-imaging data have been described elsewhere [8]. 

 Individual organs such as the heart, brain, kidney, spleen, and glands were delineated using a combination of feature detection for initial automatic segmentation and then manually correcting the results. In organs such as the ovaries, where lack of uniform color and lack of high contrast between neighboring tissues prevented automatic approaches, manual segmentation was carried out in selected sections and 2D interpolation was used to complete the set. Subsequently, each segmented region was rendered using the volume visualization techniques detailed above. Volumes of organs and tissues were computed from pixel count of 2D segmentation, the 2D pixel dimension and section thickness. The gross animal weights and brain volumes were used as normalizing factors for comparing organ volumes between the transgenic and the control. 

 For comparing specific bone lengths such as the femur, digital sections through the axis of the bone were created through interactive multiplanar reformatting of the original 3D data volume. Measurement tools available in the software package were used to compute bone length and cross-sectional areas. The length of the animal (nose to base of tail) was used as the normalizing factor for comparison across animals. Standard tools available in AMIRA were used for volume presentation such as snapshot capture, and movie making.

## 4. Results

**Figure 1:**
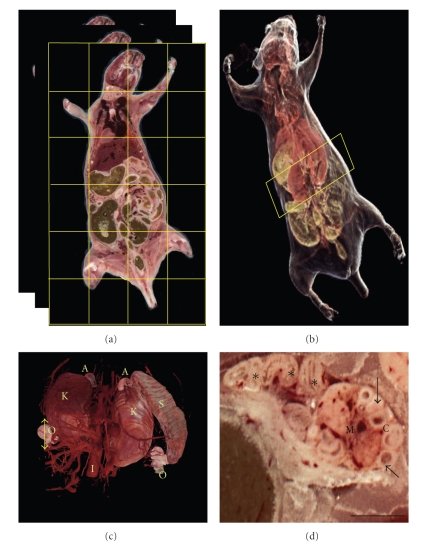
Multiscale Visualization. (a) A 421-day-old female PEPCK-C^mus^ mouse was cryo-imaged at very high-resolution by automatically tiling across the block-face and sectioning serially. (b) The 2D stitched images were converted into a 3D volume >60 GB and visualized in low-resolution through automatic enhanced volume visualization schemes. Interactively, regions were then chosen (box) to visualize in higher-resolution. (c) From the chosen region, a higher-resolution rendering shows the kidneys (K), the adrenal glands (A) above the kidneys, the spleen (S), the inferior vena cava (I), and the pair of ovaries (O). Note that all renderings are in true color. Sections were then chosen (double-arrow line) to investigate structures in full resolution. (d) Coronal section through the left ovary (section 280). The medulla region (M) consists of loose connective tissue and large blood vessels while the peripheral cortex region (C) contains a number of ovarian follicles (arrows) in different stages of development. Sections through several fimbriae (∗) of the infundibulum of the uterine tube can be seen to the left of the ovary (Bar = 1 mm).

The image acquisition and multiscale volume visualization is illustrated in Figure 1. From the stitched 2D image stack (Figure 1(a)), a 60 GB 3D volume was created and visualized at multiple resolutions. In the down-sampled whole mouse rendering, major organs were clearly seen through transparent skin (Figure 1(b)). A bounding box was placed in the abdomen area to extract the region in highest resolution and organs such as the kidneys, adrenal glands, ovaries, and vasculature segmented and reconstructed in 3D (Figure 1(c)). All volume renderings were in true color and obtained without image spatial segmentation. 2D reformatted sections were then extracted by interacting with this subvolume at will. For example, a section through the left ovary shows the inner medulla consisting of loose connective tissue and large blood vessels and a peripheral cortex region containing a number of ovarian follicles in different stages of development as small as 50 *μ*m in diameter. A few fimbriae of the infundibulum of the uterine tube can also be seen. At this full resolution, these and other many small structures like ≈50 *μ*m villi of the small intestine, mesenteric blood vessels ≈20 *μ*m in diameter, thin walls of the vessels ≈30 *μ*m and skeletal muscle and nerve fibers were very clearly seen.

**Figure 2:**
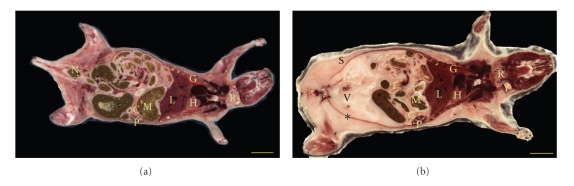
Comparison of a PEPCK-C^mus^ mouse and a control mouse. A coronal 2D section (no. 390 of 586) of the transgenic PEPCK-C^mus^ is shown in (a) while (b) shows similar coronal 2D section (no. 240 of 616) of the control mouse. Both images were composited by tiling 24 images each with an in-plane pixel size of 15.6 *μ*m. In both cases, the embedding compound OCT has been replaced with a background of black through automated image processing. Major organs like the heart (H), lungs (G), liver (L), salivary glands (R), stomach (M), spleen (P), pancreas (C), small intestine (I), colon (N), and lymph node (Y) are visible. Compared to the older PEPCK-C^mus^ mouse, the control mouse has large amounts of visceral (V) and subcutaneous (S) adipose tissue. The peritoneum (∗) of the control mouse is thus distinctly visible (Bar = 10 mm).

Coronal section images composited from 24 (= 4 × 6) individual tiles are shown in Figure 2. Images were stitched in high quality without perceived seams. Major organs like the heart, lungs, liver, salivary gland, stomach, spleen, pancreas, small intestine, colon and lymph node are visible. The abdominal regions of the two animals show extraordinary difference in adipose tissue content. The very visible peritoneum of the control mouse encases a large amount of visceral adipose tissue as compared to the PEPCK-C^mus^ transgenic mouse. The transgenic mouse also has negligible amount of subcutaneous fat. Mice were maintained on a diet of standard mouse chow. This extraordinary difference in adipose tissue depots is a general finding for these animals. Using semiautomatic segmentation techniques, we computed fractional vascularization in the visceral adipose tissue encased by the peritoneum in the lower abdomen area around the cervix. The gross adipose tissue volume, estimated from voxels in 86 sections, was 38% more in the younger control mouse compared to the PEPCK-C^mus^ transgenic mouse. The percentage of tissue classified as vasculature within this limited adipose tissue mass was higher for the PEPCK^mus^ mouse as compared to the control (2.5% vascularized in the transgenic animal; 0.4% in control). The highly vascularized pancreas extending from the spleen to the duodenum can be also seen in both the section images.

**Figure 3:**
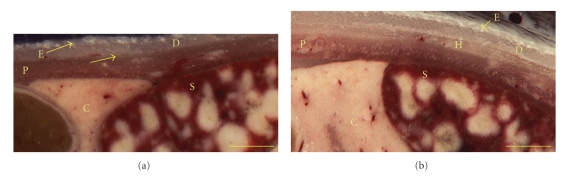
Comparison of coronal section images to estimate skin thickness. The hypodermis (H) of the older PEPCK-C^mus^ mouse in (a) is thinner than that of the control (b) by 83% while the epidermis (E) of the control is 16% thinner than the transgenic. The dermis (D) of both animals was similar. The parietal peritoneum (P) thickness was also same. The pancreas (C) and spleen (S) are visible in these images. Skin thickness as an ageing marker indicates that the ageing process may be slower in the PEPCK-C^mus^ mouse (Bar = 1 mm).

In Figure 3, coronal section images were compared for estimating skin thickness. The dermal layers were distinct with an 83% thinner hypodermis for the transgenic as compared to the control (502 mm for control and 84 mm thick for the PEPCK^mus^ mouse). The epidermis of the control was 16% thinner than that of the transgenic (407 mm transgenic, 348 mm control). The dermis was similar in thickness (578 mm transgenic, 621 mm control). The parietal peritoneum, visible below the skin, was also measured and found to be same. 

**Figure 4:**
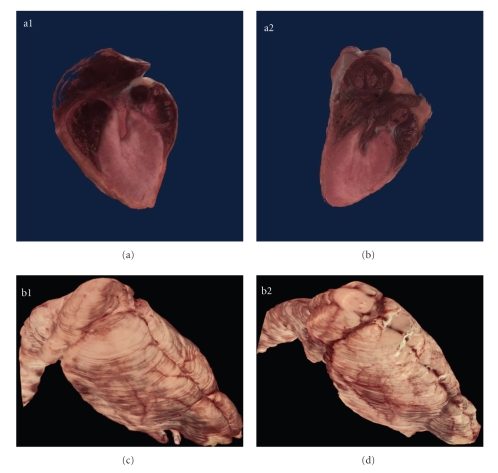
Organ visualization. Cutaway through the 3D reconstructed hearts of the PEPCK-C^mus^ (a1) and control mouse (a2) reveals the chambers of the heart, pulmonary trunk and pulmonary veins. Manual segmentation of the heart from the 2D images also enabled computation of the heart volume. The heart of the PEPCK-C^mus^ mouse is 351.52 cubic mm in volume, while the heart from the control heart animal is 366.87 cubic mm. Other organs such as the brain of the PEPCK-C^mus^ (b1) and the control mouse (b2) were also semiautomatically segmented, visualized in 3D, and volumes computed. Table 1 lists the comparative volumes. The brain volume was also used as a normalizing factor for comparison of other organ volume estimates.

Selected organs were semiautomatically segmented and visualized in 3D (Figure 4). Cutaway through the 3D reconstructed hearts of the transgenic and control mice reveal the chambers of the heart, pulmonary trunk, and pulmonary veins. Manual segmentation of the heart from the 2D images also enabled computation of the heart volume. The transgenic heart was 351.52 cubic mm in volume while the control heart was 366.87 cubic mm. The brain volume was computed and used as normalizing factor for comparing organ sizes.

**Figure 5:**
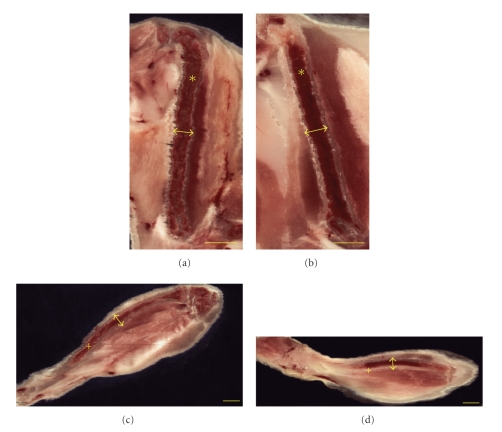
Bone Morphometry. Digital resections along the femur ((a), (b)—marked ∗) and tibia ((c), (d)—marked +) for the PEPCK-C^mus^ (a, c) and control mouse (b, d) obtained through multiplanar reformatting of the 3D data. Linear distance was computed to ascertain bone lengths. The femur measured 11.46 mm (PEPCK-C^mus^) and 12.41 mm (control) while the tibia measured 17.26 mm (PEPCK-C^mus^) and 15.12 mm (control). The tibia to femur length ratio was 24% higher for the PEPCK-C^mus^ mouse as compared to the control animal. Cortical areas were measured mid diaphyseally in cross-sections at positions indicated by arrows (Bar = 2 mm).

Multiplanar reformatting allowed bone length comparison of femur and tibia of the PEPCK^mus^ mouse and the control animal (Figure 5). Linear distance was then computed to ascertain bone lengths. The femur measured 11.46 mm (transgenic) and 12.41 mm (control) while the tibia measured 17.26 mm (transgenic) and 15.12 mm (control). The body length from tip of nose to base of tail was estimated to be 87.6 mm for PEPCK^mus^ mouse and 83.8 mm for the control mouse. When normalized to body length, the measures were similar for femur (13% for transgenic and 15% for control). However the tibia was longer for transgenic (20% for transgenic and 18% for control). The tibia to femur length ratio was calculated to be 24% higher for transgenic as compared to control. Cortical area was measured at right midtibial and midfemoral diaphysis by extracting digital planes transverse to the bone axes. Tibia cortical area was 0.74 mm^2^ for transgenic and 0.71 mm^2^ for control. Femora cortical area was 1.35 mm^2^ for transgenic and 1.38 mm^2^ for control. 

**Figure 6:**
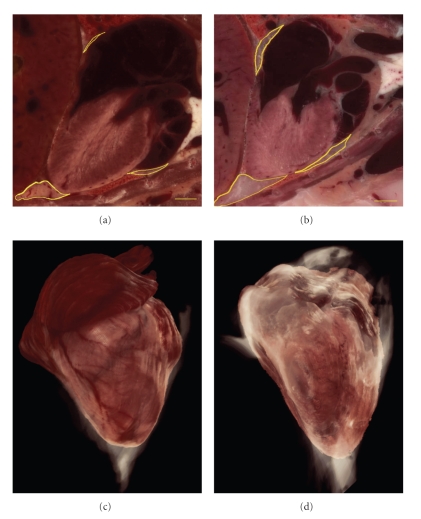
Pericardial adipose tissue. The PEPCK-C^mus^ mouse has less pericardial adipose tissue (a) compared to the control animal (b) as shown in the yellow outlines on the coronal section images. From the 2D images the heart was reconstructed in 3D with the pericardial fat rendered in white for PEPCK-C^mus^ (c) and control mouse (d). The rendering for fat was done in lower transparency so that the heart can be seen through it. Pericardial fat is considered an ageing biomarker. Note in (c) that the coronary vessels are clearly seen on the 3D surface of the heart (Bar = 1 mm).

 The pericardial fat around the heart was segmented from the 2D section images and volume visualized in 3D (Figure 6). The pericardial fat depots were more pronounced in the control mouse as compared to the PEPCK^mus^ mouse which is visible readily in the 3D volume visualization. From the 2D segmentations, volume was computed for the pericardial fat. The quantitative comparison of organ sizes is tabulated in Table 1. The major organs like brain, heart, kidney and spleen and glands such as pituitary, thymus and adrenal were segmented and volumes computed. The pericardial fat volume is also included. Both absolute values as well normalized values by gross animal weights and brain volumes are presented.

## 5. Discussion and Conclusion

The high-resolution color data volumes from the whole mouse that were obtained using the Case cryo-imaging system and multiscale viewing provide researchers the ability to seamlessly bridge the requirements of resolution and FOV. Most existing small animal imaging systems force the researchers either to scan the mouse at low-resolution to capture the entire animal in a single FOV or to choose only a region of the whole mouse for investigation at higher-resolution. As we have shown in this application, with cryo-imaging, image data is acquired at a very high-resolution, and using multiscale viewing, it is possible to view in 3D or 2D at the whole body level, zoom to a region at higher-resolution, zoom to an organ, and finally zoom to examine tissues at the highest resolution. Skeletal muscle fibers in the mouse, typically ≈45 *μ*m in diameter [23], were seen very clearly in cryo-images. In earlier work involving fluorescently labeled stem and cancer cells, we showed that one can zoom down to single cell level [7].

The multiscale approach provides an efficient way of handling extremely large whole mouse data sets. A tiled color bright field acquisition with an adult whole mouse using a 6 × 4 tiling configuration and 40 *μ*m section thickness generates >60 GB of color image data which is a prohibitively large size for volume rendering on a workstation with a conventional, single graphics processor. If fluorescence acquisition is added, it imposes an even greater burden due to increased data size. As a remedy to the extreme data problem, we designed the multiscale volume rendering approach which greatly improves the visualization experience. There are hardware considerations. Some of the important criteria to consider during multiresolution volume rendering are data access time and graphics hardware limitations on the target machine. Data access time can be greatly reduced by employing modern Solid State Disk (SSD) hard drives, which generally have higher data read and write speeds as compared to conventional hard drives. As for graphics hardware, the larger the amount of graphics RAM, the smaller the decimation that needs to be applied to produce high-resolution renderings of subregions within the large data sets.

Cryo-imaging has several advantages. First, it offers a convenient method of scanning an entire mouse since no special preparation involving stains are required. Second, a low-resolution view of the entire mouse with volume visualization of internal organs is available through automatic segmentation of OCT and automatic 3D rendering. Third, the organs and tissues are displayed in true color providing a virtual necropsy experience. Fourth, data can be seamlessly explored at every level through user-friendly select-and-view bounding boxes. Semi-automatic segmentation through algorithmic extraction of features followed by interactive manual editing provides visualization and quantification options. Multiplanar reformatting allows extraction of a digital section along arbitrary orientation. Finally, while this application has only utilized brightfield imaging, the system is capable of obtaining molecular fluorescence image data as well, thereby rendering unique versatility to the system. The featuresets of the system make it useful for applications such as anatomical phenotyping. A particularly interesting approach for identifying mutant outliers was described by Kovacevic et al. where an annotated 3D atlas of the average mouse brain with estimates of average and variability was created from MRI images, against which mutants could be compared [24]. Given the ability to segment organs in color, cryo-imaging will be well suited for such analyses for transgenic mice.

The published work on PEPCK-C^mus^ transgenic mouse contained histology of excised skeletal muscle tissue and low-resolution whole body MR imaging [22]. Cryo-imaging of this transgenic strain and its control provides the first glimpse at characterizing some of the morphological difference between the pair. The high amount of total body fat in the control, clearly visible in the 2D sections, is consistent with the published MR results. However, unlike MRI, the high-resolution of cryo-imaging allows distinction between various fat depots and color differences. As an example, we computed and compared the pericardial fat volumes and fractional vascularization in a region of the visceral fat depot in the abdomen. We were also able to detect various organs and separate the dermal layers similar to histology analysis.

It has been observed that these transgenic mice live longer and are more energetic at an older age. We chose regions and organs of the two mice to compare few standard ageing biomarkers. We note that the *older *PEPCK^mus^ mouse has less pericardial fat, less visceral and subcutaneous fat, thinner hypodermis and thicker epidermis as compared to the control animal. Skin thickness measurements are widely accepted as a biomarker of ageing while visceral and pericardial fat deposits are biomarkers of potential metabolic disorders. The thymus and spleen were chosen as ageing biomarkers since the thymus is a primary lymphoid organ considered to be consistently sensitive to morphological effects and the spleen is a secondary lymphoid organ. Characterization of thymic and splenic weights in age-related studies in nonhuman primates has shown that the thymus becomes smaller with age while the splenic changes were not statistically significant [25]. In this study, we found the older PEPCK-C^mus^ mouse has a larger thymus while the spleen is smaller than noted with the control animal. This is consistent with the inference that the transgenic mouse ages more slowly.

We also noted a difference in overall tissue coloration between the PEPCK^mus^ mouse and control. In our experience from cryo-imaging wild type mice, younger mice have an overall brighter tissue color. In this regard, the PEPCK-C^mus^ mouse looks “younger” as compared to control. For example, muscle is redder, perhaps indicating higher myoglobin content and improved circulation. Lungs are pinker, mouse indicating an enhanced circulation and oxygenation. Adipose tissue is pinker, consistent with our observation of increased vascularity. Through observations such as these, cryo-imaging can point researchers to examine certain tissues in more detail.

The PEPCK-C^mus^ mouse has a much higher exercise capacity than control [22]. A careful 3D volume measurement was carried out to compare the heart volume of this mouse with the control. While the absolute cubic volume was nearly the same, the transgenic heart volume was 40% greater when normalized by body weight. When normalized by brain volume this difference reduced. Since cardiac volume estimates derived from the cryo-images are not correlated with the cardiac phase at the time of freezing, we estimated another measure of comparison. Voxels belonging to the blood pool in the cardiac chambers and vessels in the hearts were semiautomatically identified through the color detector scheme and were excluded from cardiac volume measurement. The resulting cardiac tissue mass, when normalized by body weight, was found to be 22% more for PEPCK-C^mus^ mouse than control.

Comparison of other organ volumes such as brain and kidney and glands such as pituitary and adrenal showed a general trend of larger organs for the PEPCK-C^mus^ mouse. In order to reduce the variability due to animal size and weight, organ volume-to-animal weights were computed. To reduce the variability due to lean body mass, organ-to-brain volume normalization was used. In the case of bone lengths, the normalizing factor was body length. Digital resectioning along arbitrary planes allowed bone morphometric data to be computed from the cryo-image volume. Measurements such as length and cortical area for tibia and femur were made for two comparisons. It has been reported that short-term induced exercise in mice leads to differences in bone properties such as length and cortical area for the femur and tibia [26–28]. Further, such changes have also been recorded in ageing studies [29, 30]. The PEPCK-C^mus^ mice are highly active and can endure sustained activity at maximal speed like running on mouse treadmills [22]. The particular mouse we cryo-imaged had participated in numerous unrelated high-activity studies and was chronologically older than the control mouse. Although no longitudinal study was performed on either specimen, nonetheless, we were interested in comparing the normalized values. The PEPCK-C^mus^ mouse had a slightly higher value for tibia and lower for femora leading to a significantly higher tibia to femur ratio of 1.5. The ratio of 1.2 for the control mouse is similar to ratios derived from published reports [27]. The ratio of cortical area to bone length is considered to be indicative of bone growth [26]. This ratio was similar for both the femora and tibia for both animals perhaps suggesting that ageing does not significantly deteriorate bone biomechanics for the transgenic. 

 In this study, we have characterized the PEPCK-C^mus^ “supermouse” and demonstrated that the Case 3D Cryo-imaging system can be adopted as a morphological phenotypic screen for transgenic animals. The multiscale approach offers researchers tissue information at the most appropriate resolution traversing down the scale from mouse to organ to tissue to single cells. The ability of being able to visualize structures in 3D and extract 2D sections along any arbitrary plane makes comparisons easy and focused. Further, visualizations do not involve laborious manual segmentation and in most cases are achieved semiautomatically through the novel color feature detectors. The results of such phenotyping effort will provide a roadmap for adopting cryo-imaging for cataloging the plethora of transgenic mouse models.

## Figures and Tables

**Table 1:** Comparison of sizes of selected organs and tissues. Weight used is gross animal weight.

Organ/tissue	Volume (cu mm)		Volume/Weight (cu mm/gm)		Volume/brain volume	
	PEPCK-C^mus^	Control	PEPCK-C^mus^	Control	PEPCK-C^mus^	Control
Brain	579.65	639.20	22.467	16.910	1.0	1.0
Heart	351.52	366.87	13.625	9.706	0.606	0.574
Kidney	606.98	541.59	23.526	14.328	1.047	0.847
Spleen	105.01	125.98	4.070	3.332	0.181	0.197
Thymus	32.11	27.93	1.244	0.739	0.055	0.044
Pituitary	2.71	2.49	0.105	0.066	0.005	0.004
Adrenal	7.07	6.84	0.274	0.181	0.012	0.011
Pericardial fat	17.82	109.31	0.690	2.892	0.031	0.171
